# Data from the European registry for patients with McArdle disease and other muscle glycogenoses (EUROMAC)

**DOI:** 10.1186/s13023-020-01562-x

**Published:** 2020-11-24

**Authors:** Renata S. Scalco, Alejandro Lucia, Alfredo Santalla, Andrea Martinuzzi, Marinela Vavla, Gianluigi Reni, Antonio Toscano, Olimpia Musumeci, Nicol C. Voermans, Carlyn V. Kouwenberg, Pascal Laforêt, Beatriz San-Millán, Irene Vieitez, Gabriele Siciliano, Enrico Kühnle, Rebeca Trost, Sabrina Sacconi, Mads G. Stemmerik, Hacer Durmus, Biruta Kierdaszuk, Andrew Wakelin, Antoni L. Andreu, Tomàs Pinós, Ramon Marti, Ros Quinlivan, John Vissing, EUROMAC Consortium, Antoni L. Andreu, Ramon Martí, Tomàs Pinós, Noemi Baruch, Francisco J. Ortega, Miguel A. Martín, Carmen Navarro, Beatriz San Millán, Irene Vieitez, Andrea Martinuzzi, Monica Castelli, Federica Zucchi, Claudio Bruno, Antonio Toscano, Olimpia Musumeci, Pascal Laforêt, Sabrina Sacconi, Ros Quinlivan, Renata Scalco, Andrew Wakelin, Georgios Hadjgeorgiou, Elias Zintzaras, John Vissing, Matthias Vorgerd, Enrico Zülow, Ronald Haller, Piraye Oflazer, Hacer Durmus, Jean Pouget, Alejandro Lucia, Alfredo Santalla

**Affiliations:** 1 MRC Centre for Neuromuscular Diseases, UCL Institute of Neurology, National Hospital, London, UK; 2 Faculty of Sport Sciences, Universidad Europea de Madrid, Madrid, Spain; 3 Instituto de Investigación Hospital, 12 de Octubre (imas12), Madrid, Spain; 4 Universidad Pablo de Olavide, Seville, Spain; 5 Dept. of Conegliano-Pieve Di Soligo, IRCCS Medea Scientific Insitute, Bosisio Parini, Italy; 6 Neurology and Neuromuscular Diseases Unit, Department of Clinical and Experimental Medicine, University of Messina, Messina, Italy; 7 Department of Neurology, Donders Institute for Brain, Cognition and Behaviour, Radboud University Medical Center, Nijmegen, The Netherlands; 8 Nord/Est/Ile de France Neuromuscular Reference Center, Neurology Department, Raymond-Poincaré Teaching Hospital, Garches. AP-HP. INSERM U1179, END-ICAP, Paris Saclay University, Paris, France; 9 Pathology Deparment, Alvaro Cunqueiro Hospital, Vigo, Spain; 10 Rare Diseases and Pediatric Medicine Research Group, Galicia Sur Health Research Institute (IIS Galicia Sur), SERGAS-UVIGO, Vigo, Spain; 11 Neurology and Neuromuscular Diseases Unit, Department of Clinical and Experimental Medicine, University of Pisa, Pisa, Italy; 12 Department of Neurology, Heimer Institute for Muscle Research, University Hospital Bochum, Bochum, Germany; 13 Peripheral Nervous System and Muscle Department, CHU Nice, Université Côte D’Azur, Institute for Research On Cancer and Aging of Nice (IRCAN), INSERM U1081, CNRS UMR 7284, Faculty of Medicine, Université Côte D’Azur (UCA), Nice, France; 14 Copenhagen Neuromuscular Center, Section 6921, Rigshospitalet, University of Copenhagen, 2100 Copenhagen, Denmark; 15 Istanbul Faculty of Medicine, Istanbul University, Istanbul, Turkey; 16 Department of Neurology, Medical University of Warsaw, Warsaw, Poland; 17 Association for Glycogen Storage Disease (UK), Bristol, UK; 18 Biomedical Network Research Centre on Rare Diseases (CIBERER), Instituto de Salud Carlos III, and Research Group on Neuromuscular and Mitochondrial Diseases, Vall d’Hebron Research Institute, Universitat Autònoma de Barcelona, Barcelona, Catalonia Spain

**Keywords:** Myopathy, Rare diseases, International registry, McArdle disease, Metabolic diseases, Glycogen storage disease

## Abstract

**Background:**

The European registry for patients with McArdle disease and other muscle glycogenoses (EUROMAC) was launched to register rare muscle glycogenoses in Europe, to facilitate recruitment for research trials and to learn about the phenotypes and disseminate knowledge about the diseases through workshops and websites. A network of twenty full and collaborating partners from eight European countries and the US contributed data on rare muscle glycogenosis in the EUROMAC registry. After approximately 3 years of data collection, the data in the registry was analysed.

**Results:**

Of 282 patients with confirmed diagnoses of muscle glycogenosis, 269 had McArdle disease. New phenotypic features of McArdle disease were suggested, including a higher frequency (51.4%) of fixed weakness than reported before, normal CK values in a minority of patients (6.8%), ptosis in 8 patients, body mass index above background population and number of comorbidities with a higher frequency than in the background population (hypothyroidism, coronary heart disease).

**Conclusions:**

The EUROMAC project and registry have provided insight into new phenotypic features of McArdle disease and the variety of co-comorbidities affecting people with McArdle disease. This should lead to better management of these disorders in the future, including controlling weight, and preventive screening for thyroid and coronary artery diseases, as well as physical examination with attention on occurrence of ptosis and fixed muscle weakness. Normal serum creatine kinase in a minority of patients stresses the need to not discard a diagnosis of McArdle disease even though creatine kinase is normal and episodes of myoglobinuria are absent.

## Introduction

Understanding rare conditions is usually based on case series or small observational studies. Thus, gaining knowledge about disease phenotypes and development is challenging. For instance, lack of natural history data negatively impacts on identification of clinically important endpoints to be used in clinical trials [1]. Other challenges include delayed diagnosis, poor subject availability for recruitment into clinical trials and a lack of standardised care [1–3]. As a result, the development of new pharmacological treatments for such conditions is delayed and compromised [1, 2].

To overcome these limitations, there are increasing incentives to develop international registries. Registries are considered key instruments for developing research in the field of rare conditions, including glycogen storage diseases (GSDs) [4]. The EUROMAC project was funded by the European Union aiming to establish a network of clinical centres to develop the first European Registry for patients with McArdle Disease and very rare related muscle glycogenolytic disorders presenting with activity/exercise intolerance as the key symptom [5, 6]. The project was designed and built as described in the accompanying paper to this report [7], to raise awareness of diagnostic accuracy of muscle GSDs, improve the care, and collect important clinical and epidemiological data that can better describe the phenotypes and indicate endpoints for use in future clinical trials.

This paper describes the data collected in the EUROMAC registry for patients with McArdle disease.

## Methods

The registry was designed under the guidance and consensus of EUROMAC members at specific meetings during the first months of the EUROMAC project. EUROMAC members were twenty full and collaborating partners from eight European countries (Denmark, France, Germany, Greece, Italy, Spain, Turkey and United Kingdom) and USA. The Netherlands and Poland joined the project later and contributed patients to the registry. The registry obtained the approval of all local Institutional review Boards for patient entry at the registry website (www.registryeuromac.eu). The project was divided into eight work packages (WP) to develop the registry (see Table 2 in accompanying paper) [7]. The technical setup and data security for the registry are detailed in the accompanying paper [7].

After review by people affected by GSDs and by a patient representative (WP2 – AGSD-UK) to ensure clarity [8], the participant information sheet and consent form were translated into languages of the participating countries and adapted to follow local regulations. All patients consented in writing before inclusion in the registry.

Any doctor working at a European institution was able to register on the EUROMAC platform and enter patient data. Inclusion criteria were patients with a diagnosis of one of the 14 known muscle GSDs either verified by the presence of two pathogenic mutations in the relevant genes (one pathogenic mutation for phosphorylase *b* kinase and phosphoglycerate kinase deficiencies as they are X-linked) or for McArdle disease and phosphofructokinase deficiency, lack of enzymatic staining on muscle biopsy. Patients with Pompe disease were excluded as there is a well-established registry for this disease already (https://clinicaltrials.gov/ct2/show/NCT00231400). Following informed consent from the participant, data were uploaded onto a safe, encrypted web-based registry. Recruited participants were able to log in, review their own information and complete selected sections with their personal experiences. None of the recruited participants or participating doctors were allowed to see data from other patients. Data entry items are shown in Table 3 of the accompanying paper [7]. Muscle strength was assessed by manual muscle testing in patients older than 18 years (n = 247) by a trained neuromuscular specialist. Data was only entered once, except if missing data was uploaded later. Data was based on clinical status and medical history at data entry.

## Results

### Patient registry

**Fig. 1:**
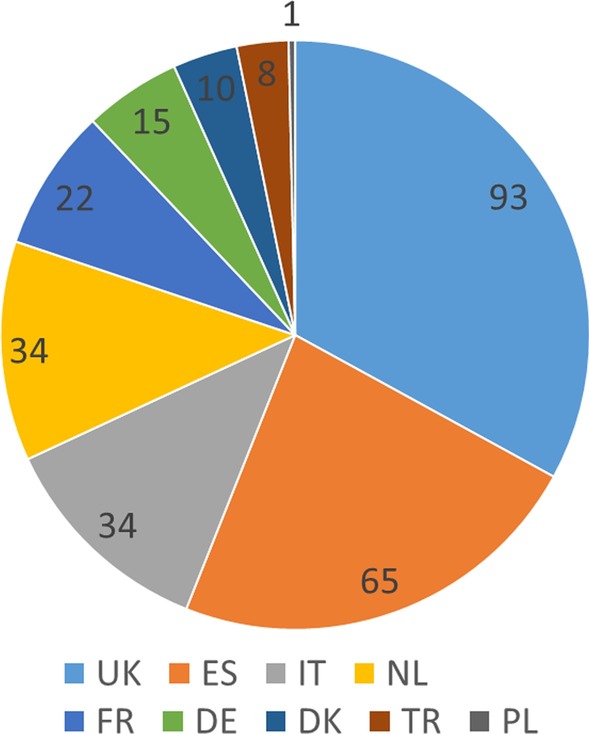
Registered patients by country. UK: United Kingdom; SP: Spain; IT: Italy; NL: Netherlands; FR: France; DE: Germany; DK: Denmark; TR: Turkey; PL: Poland

The first patient was entered into the registry in February 2015. Until March 2018, 313 patients from 10 countries were added to the registry. Thirty-one patients were excluded from data analysis due to incomplete data entry related to diagnosis (i.e., missing muscle biopsy and/or missing genetic data). Thus, 282 participants were included in data analysis (Fig. 1). In this cohort, the most frequent GSD diagnosis was McArdle disease (n = 269; 95.4%). Other conditions included GSDIIIa (n = 2), GSDVII (n = 5), GSDIX (n = 2), GSDX (n = 1), GSDXIV (n = 1), and GSDXV (n = 2). Detailed information on the 13 patients with rarer types of GSD are omitted as the sample is too small for data analysis. Following a EUROMAC teaching course held in Warsaw, the first Polish patient was diagnosed with McArdle disease, and entered into the registry [9].

### McArdle disease: GSDV (n = 269)

#### Patient demographics

Age ranged from 8–82 years, with a median age of 46 years. Fifty-three percent were males (n = 142) and 47% females (n = 127). Completeness of data entry for different items is shown in Table 1.

**Table 1:** Incomplete data related to patients with McArdle disease (GSDV)

	Missing data (n)	Total number of patients considered for the outcome analysis (n)
Current age	1	268
Age at diagnosis	3	266
BMI	44	203^a^
Second wind	41	228
CK (baseline, all)	93	176
CK (baseline/ weakness)	39	88^b^
Analgesia use	91	178
Comorbidities	28	241

^a^Only patients aged 18 years or older were included in the data analysis for body mass index (BMI)

^b^Creatine kinase (CK) levels are shown for all patients in whom it was measured at baseline and the subset of patients who were affected by muscle weakness

#### Diagnosis

The median age at diagnosis was 30 years (range: 5–79 years), with no difference between men and women. Table 2 illustrates the use of the diagnostic tests (genetic testing, muscle biopsy). In total, 90.0% of patients had a genetic test performed at some stage of the diagnostic investigation.

**Table 2:** Diagnostic investigations and age at diagnosis

Diagnostic test	n (%)	Age at diagnosis median age (age range)	Current age median age (age range)
Genetic test (only)	101(37.5%)	27.5 years (8–79)	43 years (10–80)
Muscle biopsy (only)	27 (10.0%)	30 years (12–58)	50 years (16–75)
Muscle biopsy + Genetic test	141 (52.4%)	32 years (5–76)	46 years (8–82)
Total	269		

Of all McArdle patients with two pathogenic variants identified (n = 242), the allele frequency for the c.148C>T (p.R50X) variant was (62.4%) occurring as either homozygous (108) or compound heterozygous (86). The allele frequency for the c.613G>A (p.G205S) variant was 6.4% (homozygous in 5 and compound heterozygous in 21), and the allele frequency for the c.2262delA was 4.3% % (homozygous in 4 and compound heterozygous in 13) (Table 3). All other alleles occurred at a frequency below 2%. Only five other pathogenic variants were found in 5–10 McArdle patients, and the other pathogenic variants were mostly private. Sixteen novel pathogenic variants, not previously reported in the Human Gene Mutation Database, were identified (Table 3).

**Table 3:** List of pathogenic variants assessed in 242 patients with McArdle disease

Mutation	Heterozygous	Homozygous
c.148C>T	86	108
c.613G>A	21	5
c.2262delA	13	4
c.1190T>C	8	0
c.808C>T	6	0
c.1A>G	1	5
c.2392T>C	7	0
c.1466C>G	4	0
c.1282C>T	3	0
c.13_14delCT	3	0
c.1129A>T	2	0
c.1162_1169delTGGCCGGTinsA	2	0
c.1366G>A	2	0
c.1468+1G>A	2	0
c.1760T>C	2	0
c.1769G>A	1	1
c.1827G>A	2	0
c.2056G>A	2	0
c.2113_2114delGG	2	0
c.2128_2130delTTC	0	2
c.2143C>T	2	0
c.2385_2386delAA	2	0
c.415C>T	1	1
c.481C>T	2	0
c.661-601G>A	1	1
c.1345G>A	2	0
c.1768+1G>A	2	0
*c.2259_2261delA*	*1*	*1*
*c.1275-1276insG*	*2*	*0*
*c.2177*+*1G>A*	*2*	*0*
c.773-2A>T	1	0
c.1463C>T	1	0
c.1723A>G	0	1
c.1730A>G	1	0
p.R387Afs*37^θ^	1	0
c.1805G>A	1	0
c.1948C>T	1	0
c.2075_2076delCCinsAAA	1	0
c.2083G>A	1	0
c.2441G>A	1	0
c.244-3_244-2delCA	1	0
c.255C>A	1	0
c.347T>C	1	0
c.458T>G	1	0
c.580C>T	1	0
c.715_717delGTC	1	0
c.1093-1G>T	1	0
c.1239+1	1	0
c.397G>A	1	0
c.1475G>A	1	0
c.1094C>A	1	0
*c.1093-2A*>*G*	*1*	*0*
*c.1275delG*	*1*	*0*
*c.507G*>*T*	*0*	*1*
*c.558delC*	*1*	*0*
*c.345*+*2T*>*A*	*1*	*0*
*c.597-598delT*	*1*	*0*
*p.V25fs51X*^*θ*^	*0*	*1*
*c.1477delC*	*1*	*0*
*c.227delG*	*1*	*0*
*c. 2178*+*1G*>*A*	*1*	*0*
*c.1478T>C*	*0*	*1*
*c.1700A>C*	*0*	*1*
*c.1700A>G*	*1*	*0*

Pathogenic variants are listed according to their frequency. Numbers in columns below the headings “heterozygous” and “homozygous” show number of persons carrying the pathogenic variant in a compound heterozygous or homozygous state. Pathogenic variants that are in italic are novel pathogenic variants not present in Human Gene Mutation Database. θ signifies frameshift pathogenic variants where the nucleotide change is unavailable

#### Body mass index (BMI)

**Fig. 2:**
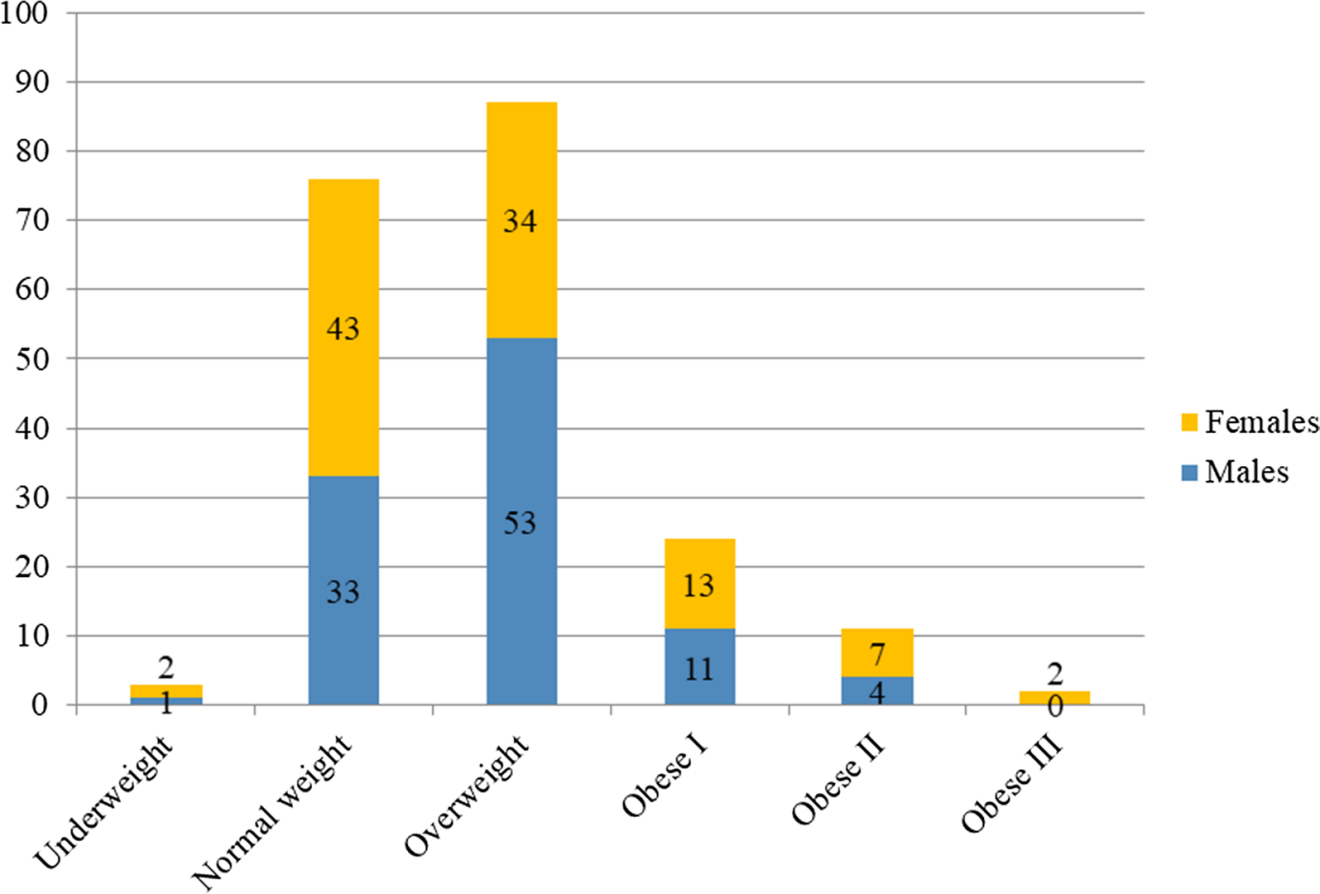
Body mass index (BMI) in 203 adult patients with McArdle disease (GSDV). Underweight: < 18.5; Normal weight: 18.5–24.9; Overweight: 25.0–29.9; Obese I: 30.0–34.9; Obese II: 35.0–39.9; Obese III: ≥ 40 kg/m^2^

BMI was analysed in people older than 18 years (n = 203). The median BMI in this cohort was 25.9 kg/m^2^ (range: 16.6–52.8 kg/m^2^). Abnormal BMI was observed in 62.6% (Fig. 2).

#### McArdle-related signs and symptoms

Most patients identified spontaneous *second wind* (82%, n = 187), while 41 patients (18%) had not previously recognised the phenomenon. Baseline serum creatine kinase (CK) levels varied from 99 to 32,394 IU/L (median: 1,475 IU/L). Twelve McArdle patients (6.8%) had baseline CK below the normal upper reference level of 240 IU/L. Ten patients (5.7%) had CK levels higher than 10,000 IU/L. Previous episodes of myoglobinuria were reported by 61.7% of patients (n = 166). 71.1% of these patients had recurrent rhabdomyolysis. No previous episode of myoglobinuria was reported by 39.4% of patients (n = 106).

Muscle weakness was observed in 127 (51.4%). The median age of those patients was 47 years. Weakness affected men (52%) and women (48%) equally. Weakness preferentially affected upper body and trunk muscles as shown in Table 4. The median age of patients not affected by muscle weakness was also 47 years.

**Table 4:** Muscle weakness in 127 adults with McArdle disease

Affected muscles	n
Upper limbs/trunk	74
Lower limbs	21
Upper and lower limbs + trunk	31
Ptosis	8
Mastication muscles	1

The total number of patients in the table exceeds 127 as 7 of 8 patients with ptosis and the patient with affection of the masticatory muscles also had limb or truncal weakness. Weakness in upper and lower limbs and trunk, was defined as weakness of one or more muscle groups in each region

There were no significant differences in CK levels in patients with weakness compared with those with no weakness (Table 5) although five of the patients with weakness had CK lower than 240 IU/L. Eight patients (3.2%) had ptosis (median age: 54.5 years) and in one of them, ptosis was the only evidence of muscle weakness. Relationship between genotype and laboratory/clinical findings can be viewed in Additional file 1: Table S1.

**Table 5:** Serum creatine kinase (CK) levels in patients according to muscle weakness

	Median CK (IU/L)	Range (IU/L)
Weakness	1519.5	137–32,394
No weakness	1271	99–25,459

Forty-two patients (23.6%) used analgesic medication to manage pain symptoms, which included: paracetamol (n = 19), nonsteroidal anti-inflammatory drugs (n = 17), opioids (n = 14), central analgesic (n = 5) and steroids (n = 1).

#### Cardiorespiratory fitness (peak oxygen uptake [VO_2peak_])

A cycle ergometry exercise test was performed in 71 patients in Spain (n = 41), Italy (n = 14), United Kingdom (n = 9) and Denmark (n = 7); one exercise test was excluded from data analysis as the cycle test was interrupted prematurely. The median VO_2peak_ was 18.5 ml/kg/min (7–33). This value is a little less than half that expected in matched healthy people. The lowest result was seen in a 48-year-old woman with fixed muscle weakness.

#### Comorbidities

In this cohort, 80 people (33.2%) did not have any comorbidity (median age: 34 years). Recorded comorbidities are shown in Table 6. Median age of patients with comorbidities was 50 years (12–82). Hypothyroidism affected men (n = 7) and women (n = 7) equally (age range at data entry: 17 – 77 years; median age: 59.5 years; mean age: 52.5 years). The two thyroid gland tumors reported were a benign adenoma and a malignant carcinoma. Ophthalmological conditions included retinal problems (n = 3), cataract (n = 1) and uveitis (n = 1). Retinal problems included macular degeneration, retinoschisis and a history of three retinal detachments, and retinal dystrophy. Of the 19 patients with a history of acute kidney failure, 17 had a positive history for a previous episode of myoglobinuria. Thyroid problems (n = 20) were the most frequent among the endocrine diseases, with prevalence higher than diabetes (n = 15).

**Table 6:** Comorbidities reported

Comorbidities	N	Frequency in this Cohort (n = 241) (%)	Median age (range) Years
Hypertension	41	17	57 (39–82)
Endocrine disease (Total)	38	15.7	56 (17–77)
Diabetes	15		
Hypothyroidism	14		
Thyrotoxicosis	2		
Thyroid tumour	2		
Hyperthyroidism	2		
Other endocrine problems	3		
Musculoskeletal/rheumatic disease	31	12.9	52 (15–82)
Coronary artery disease	20	8.3	62 (42–80)
Other cardiovascular disease	10	4.1	54.5 (35–80)
Hyperuricemia/gout	28	11.6	60.5 (24–80)
Gastroenterological disease (Total)	27	11.2	52 (12–77)
Gall bladder problems	6		
Hiatus hernia and/or acid reflux	4		
Crohn’s disease	3		
Irritable bowel syndrome	3		
Coeliac disease	3		
Diverticulitis or polyposis	2		
Constipation	1		
Gastritis	1		
Liver disease^a^	4^a^		
Pancreatitis^a^	2^a^		
Neurological disease	24	10	51.5 (13–81)
Respiratory disease	23	9.5	51 (12–75)
Acute renal failure	19	7.9	53 (33–76)
Chronic renal failure	3	1.2	44 (17–49)
Dyslipidaemia	21	8.7	57 (24–82)
Mental disorder (Total)	16	6.6	49 (13–75)
Depression	9		
Anxiety	4		
Bipolar disorder	1		
Schizophrenia	1		
Post-traumatic stress disorder	1		
Cancer	11	4.6	55 (33–82)
Anaemia/hyperbilirubinemia	9	3.7	39 (26–82)
Ophthalmological disease	5	2.1	67 (60–73)
Others	54	22.4	53 (19–80)

^a^Two patients had two gastroenterological diseases: Thus, the sum in the table is higher than the total number of patients affected by a gastroenterological disease

## Discussion

Besides a specific international Pompe disease registry, EUROMAC is the only international registry of muscle glycogenoses reported to date that serves as a pan-European reference to register people with rare muscle GSDs to improve knowledge and promote research in these conditions. Besides unravelling phenotype characteristics of McArdle disease as discussed below, the EUROMAC project also played a key role in education and training in several European countries. The registry has also contributed to the promotion and/or implementation of international clinical trials [10–21], illustrating its positive role in promoting translational research.

The most common muscle GSD represented in this European cohort was McArdle disease.

Although data on age at onset of symptoms were not recorded in the registry, because of a presumed high level of recall bias, there seems to be a considerable delay in diagnosing the GSDs, which highlights the need for better awareness of these conditions. So, besides creating the EUROMAC registry, it emphasizes the importance of the EUROMAC education and dissemination activities [22–25]. The increased use of genetic diagnostic testing reflects the shift towards a molecular genetic approach across all participating countries, which likely will facilitate more timely diagnosis of muscle GSDs. The registry confirmed the high prevalence of the two common variants, c.148C>T (p.R50X) and c.613G>A (p.G205S) in the European population of McArdle patients, and added 16 novel pathogenic variants to the list of approximately 150 known, pathogenic *PYGM* variants.

As previously reported, the serum CK levels were high in most patients with McArdle disease, however, it was surprising to find that 6.8% of patients had CK levels within normal range. All patients with normal CK levels had a confirmed genetic diagnosis and had typical symptoms of McArdle disease, such as exercise-induced pain and contractures and exercise intolerance, which led to diagnostic testing for the disease. Four of the patients with normal CK levels had a history of myoglobinuria.

Normal CK levels were also seen in more severely affected people, including those with fixed muscle weakness. An important message therefore is that a normal CK and a negative history for myoglobinuria do not exclude a diagnosis of McArdle disease.

There was a higher frequency of fixed muscle weakness reported in this cohort compared with previous studies of patients of the same age [23, 24, 26], suggesting that this condition may have been under-recognized in the past. A major new finding from our registry data was the presence of ptosis in a small proportion of patients with McArdle disease, suggesting that this can also be a feature of this glycogenosis, as it has also been noticed in patients with GSDII (Pompe disease) [27, 28].

We also found that BMI was higher than normal in almost two thirds of patients, likely reflecting a sedentary lifestyle related to the poor exercise tolerance and muscle pain on exertion. This is relevant for these diseases as overweight and general low physical activity level promotes low insulin sensitivity, diabetes and metabolic syndrome, factors that may exacerbate the condition. Coronary artery disease was the most common heart disorder. Surprisingly, thyroid disease was the most common endocrine disorder, with a higher frequency than diabetes. The prevalence of hypothyroidism is 1–2% in iodine-sufficient parts of the world [29], which is lower than the prevalence reported in the present study (5.8%). This is the first time an increased frequency of thyroid problems was reported in people affected by McArdle disease, but the reason for this warrants further study. The authors recommend performing regular TSH surveillance for patients at their follow-up visits.

Three patients had chronic renal failure, indicating that recurrent rhabdomyolysis, which is common in this population, rarely results in irreversible kidney damage. Three patients had retinal problems. Even though a recently published case series has identified four more McArdle patients with retinal disease [30], it is still uncertain whether this is part of the disease. The possibility of associated retinal disease should be explored further, and therefore a retinal exam should be performed at least once in patients.

Limitations of this study include the collection of retrospective data and the presence of missing data. Further efforts to maintain a prospective registry for rare diseases may help gaining further understanding on the natural history of rare conditions such as McArdle disease.

## Conclusions

The EUROMAC project and international registry have significantly raised awareness of McArdle disease, and should inspire opinion leaders in other fields to develop similar patient registries.

The analysis of the registry data has given insight into new phenotypic features of McArdle disease and the variety of co-comorbidities affecting people with McArdle disease, which should lead to strategies reducing and managing these in the future, including controlling weight, and preventive screening for thyroid and coronary artery diseases. The data of the registry suggest that physical examination should focus more on occurrence of ptosis and fixed muscle weakness, which may be overlooked, and stresses the need not to discard a diagnosis of McArdle disease even though CK is normal and episodes of myoglobinuria are absent.

## Supplementary information

**Additional file 1**. Genetic variants according to clinical features.

## Data Availability

Nearly all data relevant to the study is included in this article. Other data are available upon reasonable request, if ethic committees involved grant permission to do so.

## Abbreviations

AGSD-UK: Association for Glycogen Storage Disease (UK)
CK: Creatine kinase
GSD: Glycogen storage disease
GSD-V: Glycogen storage disease type V
VO_2peak_: Peak oxygen uptake
NSAID: Non-steroidal anti-inflammatory drugs
WP: Work package
